# Koala retrovirus viral load and disease burden in distinct northern and southern koala populations

**DOI:** 10.1038/s41598-019-56546-0

**Published:** 2020-01-14

**Authors:** Nishat Sarker, Jessica Fabijan, Helen Owen, Jennifer Seddon, Greg Simmons, Natasha Speight, Jasmeet Kaler, Lucy Woolford, Richard David Emes, Farhid Hemmatzadeh, Darren J. Trott, Joanne Meers, Rachael Eugenie Tarlinton

**Affiliations:** 1 School of Veterinary Science, The University of Queensland, Brisbane, Australia; 2 School of Animal and Veterinary Sciences, The University of Adelaide, Adelaide, Australia; 3 School of Veterinary Medicine and Science, University of Nottingham, Nottingham, United Kingdom; 4 Advanced Data Analysis Centre (ADAC), University of Nottingham, Nottingham, United Kingdom; 5 Laboratory Sciences & Services Division, International Centre for Diarrhoeal Disease Research, Dhaka, Bangladesh

**Keywords:** Genetics, Molecular biology

## Abstract

Koala retrovirus (KoRV) displays features of both an endogenous and exogenous virus and is linked to neoplasia and immunosuppression in koalas. This study explores the apparent differences in the nature and impact of KoRV infection between geographically and genetically separated “northern” and “southern” koala populations, by investigating the disease status, completeness of the KoRV genome and the proviral (DNA) and viral (RNA) loads of 71 northern and 97 southern koalas. All northern animals were positive for all KoRV genes (*gag*, *pro-pol* and *env*) in both DNA and RNA forms, whereas many southern animals were missing one or more KoRV genes. There was a significant relationship between the completeness of the KoRV genome and clinical status in this population. The proviral and viral loads of the northern population were significantly higher than those of the southern population (P < 0.0001), and many provirus-positive southern animals failed to express any detectable KoRV RNA. Across both populations there was a positive association between proviral load and neoplasia (P = 0.009). Potential reasons for the differences in the nature of KoRV infection between the two populations are discussed.

## Introduction

Koala retrovirus (KoRV) was first identified in tissues from a leukemic koala in 1988^1^ and the full genome sequence was published in 2000^2^. This is a unique endogenous retrovirus which is also capable of being transmitted horizontally^3^. It is thought that KoRV plays a role in the pathogenesis of lymphoid neoplasia as well as causing immunosuppression, making koalas more susceptible to developing overt chlamydial disease^4^, which is a major threat to koala populations. However while there appears to be a strong association between KoRV infection and these diseases in koalas, there is currently little if any published data proving a causal link^3,5,6^.

Anecdotally, there are differences in disease prevalence between koala populations in northern and southern Australia. The prevalence of lymphoid neoplasia is high in northern koalas^7^ compared to southern koalas where lymphosarcoma has only recently been reported^8^. The prevalence of *Chlamydia pecorum* infection and overt chlamydial disease is also high in northern koalas in comparison to southern populations, with chlamydial disease reported in 52% of more than 20,000 koala admissions to wildlife hospitals in South East Queensland (SE QLD)^9^. In contrast, a recent necropsy study in South Australia (SA) reported only 21% of koalas with clinically overt disease^10^. Conversely, SA kocalas have a higher prevalence of oxalate nephrosis than QLD koalas, with 55% of SA koalas demonstrating renal dysfunction due to oxalate nephrosis^11^.

These differences in disease prevalence may be the result of genetic differences between northern southern koala populations^12^. Animals in the southern part of the range (the states of Victoria, South Australia [SA] and southern New South Wales [NSW]) have undergone a severe genetic bottleneck as a result of hunting pressures in the late 1800’s. These populations were restocked across much of their former population range from a very small number of island populations and as such fall into a separate genetic lineage compared to northern animals (Queensland [QLD], northern NSW)^13^. It is also conceivable that differences in KoRV parameters may be contributing to the variation in disease prevalence between northern and southern populations.

The literature to date has indicated that koala populations in QLD exhibit 100% prevalence of KoRV, while a lower prevalence in southern koalas has been reported, with only 25% of wild Victorian koalas testing positive for KoRV provirus^14,15^. These studies determined prevalence based on the presence of the KoRV proviral *pol* gene^14,15^. In contrast, the transcriptomic analysis of QLD and SA koalas submandibular lymph node tissue demonstrated KoRV transcripts in 100% of animals from both populations^16^, although 27.6% SA koalas were missing transcripts of *pol* or *env* or both of these genes and had only truncated transcripts of the *gag* gene^16^. Given these complex findings, the role of KoRV in causing differences in disease occurrence between northern and southern populations is unlikely to be due only to proviral prevalence, and other viral characteristics must be explored. Investigating these characteristics in both populations may shed light on the pathogenesis of KoRV. It is possible that the full length of the proviral genome is present but not actively transcribed in southern animals, it is also possible that the provirus is fragmented or truncated and lacks the ability to transcribe accurately. The levels of KoRV proviral DNA load and viral RNA load also may influence disease expression in individual koalas. Viral RNA load has been shown to be a significant predictor for disease progression in other retroviral diseases such as HIV-1^17^ and Feline immunodeficiency virus (FIV) infections^18^. With respect to KoRV, koalas with neoplasia exhibited significantly higher levels of plasma viral RNA load compared to koalas without neoplasia^19^.

This study aimed to investigate characteristics of KoRV infection in koala populations in QLD (representative of the northern genotype) and SA (representative of the southern genotype). Results from a comprehensive suite of PCRs including conventional PCR, qPCR and RT-qPCR were analysed to obtain a greater understanding of KoRV provirus (DNA) and virus (RNA) in the two populations. The study aimed to: (i) investigate the completeness of KoRV provirus and virus genomes, (ii) characterise differences in KoRV proviral load and viral load in the two populations and (iii) investigate associations between KoRV completeness, proviral load, viral load and disease.

## Methods

### Sample collection and preparation

Samples were collected from wild-rescued koalas euthanased for clinical reasons and submitted for post-mortem examinations from South East QLD (Greater Brisbane region) and SA (Mount Lofty Ranges). Clinically healthy northern captive koalas were also sourced from Sea World Paradise Country (SWPC), Australia. Blood was collected into EDTA tubes. Ethical approval for this study was granted by the University of Queensland (UQ) Animal Ethics Committee, permit number ANFRA/SVS/461/12 and ANRFA/SVS/445/15, the Queensland Government Department of Environment and Heritage Protection permit number WISP11989112, University of Adelaide Animal Ethics Committee permit number S-2013-198 and South Australian Government Department of Environment, Water and Natural Resources Scientific Research Permit Y26054. All methods were performed in accordance with the relevant guidelines and regulations.

DNA was extracted from 100 µl EDTA whole blood using Qiagen DNeasy Blood & Tissue Kit according to manufacturer’s (Qiagen) instructions and finally resuspended in 200 μl elution buffer provided in the kit. The extracted DNA concentration was measured using a Nanodrop spectrophotometer at 260 nm optical density to ensure DNA quantity and quality.

A 1–2 ml aliquot of blood was centrifuged at 3000 g for 5 mins and 200 μl of the plasma was removed and added to 300 μl of RNAlater stabilisation agent (Qiagen) within 1 hour of blood collection. Plasma samples were transported to UQ and stored at −80 °C until RNA extraction. RNA was extracted using Qiagen QIAmp Viral RNA mini kit with on-column Qiagen RNase free DNase steps. The extracted RNA samples were stored at −80 °C until required.

### Clinical details and disease categories

Each koala’s sex, age determined by tooth wear, body condition score, medical history and rescue details were recorded at the time of sampling. A clinical and or post mortem examination was performed by the veterinarian or pathologist, if warranted, this included an abdominal ultrasound. Age was determined by dentition and the amount of wear on the upper premolar^20^. Tooth wear scores used to reflect age varied from 1–8 and koalas were grouped as follows: juvenile (TWC 1), young adult (TWC 2 or 3), adult (TWC 4, 5 or 6) and old (TWC 7 or 8). Body condition score of the koalas was also scored between 0–5 with ≤2 being “poor”, 3 being “good” and ≥4 being “very good”.

Animals were grouped into five disease categories based on their clinical status and post mortem results. Animals free of any clinical signs were grouped as “healthy”, these included animals euthanased due to traumatic injury and live captive animals. Animals with any neoplastic changes were grouped as “neoplasia”. Animals with signs consistent with chlamydiosis such as cystitis (based on urine staining, rump wetness, clinical and post-mortem examination), conjunctivitis, paraovarian cysts, prostatitis, urethritis, keratitis and vaginitis^21^ were grouped as “chlamydiosis”. Animals with renal pathology consistent with oxalate nephrosis^11^ were grouped as “oxalate nephrosis”, and remaining clinical presentations were grouped as “miscellaneous”.

### Determining presence and extent of KoRV genomic DNA and plasma RNA

#### Screening of DNA and RNA quality

The extracted DNA and RNA quality was initially assessed with an end point PCR of the housekeeping gene *β-actin* using published *β-actin* primers (Meers *et al*., 2014). DNA and RNA samples were amplified using Qiagen HotStartTaq Plus Master Mix kit and Qiagen OneStep RT-PCR kit respectively, following manufacturer instructions, with 35 cycles of amplification and an annealing temperature of 52 °C. Samples failing to amplify *β-actin* were excluded from further study.

#### Conventional PCR and conventional reverse transcriptase PCR

**Figure 1:**
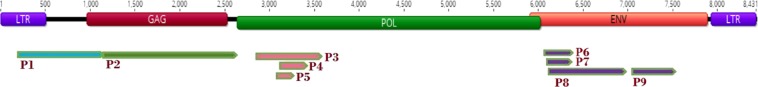
Location of target sequence for the conventional PCR and qPCR assays used in the study. PCR products (P) 1, 2, 6, 7, 8 & 9 are conventional PCR, P3 & P4 form a nested PCR; P5 is qPCR. PCR product coordinates are with respect to full KoRV genome sequence (8431 bp) (Genbank accession: AF151794).

The presence of KoRV LTR, *gag*, *pol* and *env* provirus and viral RNA were assessed using multiple primers covering different or overlapping sections of each gene (Table 1). The details of primers are depicted in Table 1 and the amplicon locations are indicated on the KoRV full genome shown in Fig. 1. Primers were designed using the full genome of KoRV-A (accession no. AF151794.2) as a reference sequence and NCBI Primer-BLAST, a primer designing tool.

**Table 1:** PCR primers and probe used in this study.

Coded	region	Forward	Reverse	Annealing temperature (°C)	Reference
P1	LTR	ACCCCGGACTTATGCAAACA	CTCACCCTGTCCCATTCTGG	52	this study
P2	*gag*	ATGGGACAGGGTGAGTCG	TCACCCAGGGTCAGGACATT	55	this study
P3	*pol*	CCTTGGACCACCAAGAGACTTTTGA	TCAAATCTTGGACTGGCCGA	50	^3^
P4	*pol*	TCATGGCTCCAACTCTTTCC	TACCAGAATCCCCAAATCCA	50	^15^
P5	*pol*	TTGGAGGAGGAATACCGATTACAC	GCCAGTCCCATACCTGCCTT	60	^19^
P6	*env*	TCCTGGGAACTGGAAAAGAC	GGGTTCCCCAAGTGATCTG	52	^4^
P7	*env*	TCACCAACCCATGACTCTCA	AGGACTCGAGACCGGCTA	52	this study
P8	*env*	GGTCCATGCTTCTCATCTCA	AGATGGAGTACTAGGGGCCG	52	^41^
P9	*env*	GCCCTCGGCCCTCCTTATTA	GGCAATCTGGAGGCTAGTCAA	52	this study
Probe	*pol*	5′-FAM-TCGACCCGTCATGGC-MGBNFQ-3′			^19^

For the *pol* gene, a nested PCR using published internal primers^15^ was used on samples that failed to produce the expected 523 bp amplicon with primer pair P3 (pol long). PCR product (1.0 μl) from the pol long amplification was used as a template for the nested PCR. Four different primer pairs were used to identify the presence of *env* gene. Proviral DNA and viral RNA was amplified using a Qiagen HotStartTaq Plus Master Mix kit and Qiagen OneStep RT-PCR kit, respectively following the manufacturer’s conditions with modification in annealing temperature and 35 cycles of amplification. Each primer pairs annealing temperature is illustrated in Table 1.

All amplified PCR products were electrophoresed in a 1.5% agarose gel/Sodium Borate buffer and 5% Syber safe stain (ThermoFisher Scientific) and then visualized in Biorad Gel Documentation system. PCR reactions were directly purified with ExoSAP-IT (Thermo Fisher Scientific), following manufacturer’s instruction.

Amplicons from PCR purified products were Sanger sequenced using multiple internal primers or sequence specific primers by Big Dye Terminators by The Animal Genetics Laboratory, University of Queensland. Sequences were then subjected to BLAST analysis through the NCBI database to determine the percentage of homology.

### Quantification of KoRV *pol* gene

Real-time PCR (qPCR) and reverse transcriptase qPCR (RT-qPCR) for the *pol* gene was used in addition to the conventional PCRs in order to increase the sensitivity of screening for KoRV provirus DNA and viral RNA, respectively and to determine the proviral load (KoRV DNA copies/10^3^ *β-actin* copies) and viral load (KoRV RNA copies/ml plasma). Only the *pol* gene was analysed with this methodology due to the conserved nature of the gene and its widespread use for KoRV diagnosis. The previously published real time PCR primers (P5 in Table 1; Fig. 1) and probe for KoRV *pol* and the housekeeping gene, *β-actin* were used for the multiplex PCR reaction^19,22^. The samples were run in triplicate on a BioRad CFX 96 system. Serially diluted DNA and RNA standards were used in each run to determine the copy number in each sample.

For the proviral DNA qPCR, a Taqman gene expression master mix (Applied biosystem) was used with the following composition: Taqman master mix 12.5 μl, *β-actin* probe 5 μM 1.25 μl, MGB *pol* probe 5 μM 1.25 μl, MGB *pol* primers forward and reverse 10 μM 2.5 μl, *β-actin* primers forward and reverse 10 μM 2.5 μl and sample DNA 5 μl. Cycling conditions were as per manufacturer’s instructions. If both *β-actin* and *pol* showed a CT value of at least 35 then the samples were considered KoRV-positive, *β-actin* positive *pol* negative samples were considered KoRV-negative. If both *β-actin* and *pol* were negative, then the sample was removed from further analysis.

Reaction mix for the RT-qPCR consisted of 1x reaction mix, 0.05 U/µl of SuperScript® III RT/Platinum® Taq Mix (SuperScript® III One-Step RT-PCR System with Platinum® Taq DNA Polymerase, Invitrogen), 10 µM of each primers (forward and reverse), 10 µM of probe, 5.5 µl of ultrapure water, and 5 µl of template. Cycling conditions were: reverse transcription at 50 °C for 30 min, Superscript platinum taq activation at 95 °C for 2 min, followed by 45 cycles of denaturation at 95 °C for 15 sec and annealing at 60 °C for 30 sec.

### Statistical analysis

Descriptive statistics were explored using Stata 14 (Mann-Whitney U for continuous variables or Chi squared for categorical variables) (Statacorp, USA) to compare proviral load and viral load between QLD and SA koalas and investigate their association with age, sex, body weight and body condition score and disease category. As proviral load and viral load were non-parametrically distributed, values were transformed to log scales for model building. After univariable analysis, a multivariable linear regression model was built in STATA 14 (Statacorp, USA) with the input variables of: location or origin (SA, QLD), sex (Male, Female), age group (juvenile, young adult, adult and old), Body condition score (poor, good, and very good), Disease category (healthy, neoplasia, chlamydiosis, oxalate nephrosis and miscellaneous) and the outcome parameter of proviral load. P-values of <=0.05 was considered significant.

## Results

### Epidemiological characteristics of collected samples

Overall, 176 koalas from QLD (n = 71) and SA (n = 105) were sampled in this study, part of the Koala retrovirus pathogenesis project, conducted between 2014 and 2017. Not all koala samples were available for all assays because of limitations on sample quantity or the quality of extracted DNA or RNA. A total of 155 animals had complete data for all parameters in the model and were included in the model.

The summary of demographic characteristics of the collected samples are described in Table 2. In general, koalas collected from QLD were older (Chi squared P = 0.0007), lighter weight (Mann-Whitney U P < 0.001) and had a lower body condition score (Chi squared P < 0.001). There were no differences in the sex ratio between the two populations. Large differences in disease prevalence were observed between the populations (Chi Squared P = 0.003); a higher proportion of QLD koalas presented with neoplasia and chlamydiosis than SA koalas, and no QLD koalas had oxalate nephrosis (Table 2). Some koalas presented with multiple conditions (eg. neoplasia and chlamydiosis). The complete data for each koala are presented in Supplementary Table 1.

**Table 2:** Demographic data summary for animals from the two populations.

Variable^a^	QLD	SA	Statistical test
**Sex**			
Female	31 (41%)	40 (40%)	
Male	44 (59%)	61 (60%)	
Total	75 (100%)	101 (100%)	Chi Squared Not Significant
**Age Group**			
Juvenile	5 (7%)	6 (6%)	
Young Adult	15 (21%)	44 (46%)	
Adult	44 (63%)	45 (47%)	
Old	6 (9%)	0 (0%)	
Total	70 (100%)	95 (100%)	Chi Squared (P = 0.007)
**Mean Weight**^**c**^			
	5.5 kg	7.7 kg	Mann Whitney U (P < 0.0001)
**Body Condition Score**			
Poor	40 (58%)	22 (22%)	
Good	18 (26%)	42 (42%)	
Very good	11 (16%)	36 (36%)	
Total	69 (100%)	100 (100%)	Chi Squared (P < 0.001)
**Clinical Status**^**b**^			
Chlamydiosis	35 (50%)	42 (40%)	
Neoplasia	9 (13%)	4 (4%)	
Oxalate nephrosis	0 (0%)	23 (22%)	
Miscellaneous	8 (11%)	11 (10%)	
Healthy	22 (31%)	29 (28%)	Chi Squared (P = 0.003)
**Total**^**a**^	**71**	**105**	

^a^Not all animals had complete data sets available.

^b^Some animals had more than one disease state so the % add up to more than 100.

QLD: Queensland; SA: South Australia.

^c^Untransformed variables.

### Status of KoRV in QLD and SA koalas

Both conventional and qPCR techniques were applied to determine the presence of various KoRV genes in the QLD and SA koalas. For detection of proviral DNA there were eight conventional PCRs and one qPCR (*pol* gene only), and for viral RNA detection there were seven RT-PCRs and one RT-qPCR (*pol* gene only). All QLD koalas (n = 71) were positive in all PCRs for both KoRV proviral DNA and viral RNA. In the SA koalas, there were varying levels of positivity in the KoRV proviral DNA conventional PCRs, ranging from 41% (40/97) in the *env* P9 PCR to 94% in the *env* P7 PCR (Table 3). All but one SA koala were provirus positive in the qPCR (P5), i.e. 96/97 (99%). When the PCRs (including qPCR) were grouped by KoRV gene (and classing LTR as a gene), 77/97 koalas were positive for all 4 genes, 19/97 were positive for only 1, 2 or 3 genes and one koala was not positive for any gene (detailed in Supplementary Table 2).

**Table 3:** Rate of PCR positivity for multiple conventional and qPCR reactions for KoRV proviral (DNA) and viral (RNA) in SA koala samples.

	Gene: Primer Pair	Provirus DNA	Viral RNA
Conventional PCR and RT-PCR	LTR: P1	89/97 (92%)^a^	N.D.
	Gag: P2	79/97 (81%)	5/41 (12%)
	Pol: P3	37/97 (38%)	5/41 (12%)
	Pol nested: P4	40/77^b^ (52%)	1/36^b^ (3%)
	**Total** ***pol*** **positive**^**c**^	77/97 (79%)	6/41 (15%)
	Env: P6	88/97 (91%)	10/41 (24%)
	Env: P7	91/97 (94%)	9/41 (22%)
	Env: P8	40/97 (41%)	5/41 (12%)
	Env: P9	63/97 (65%)	5/41 (12%)
	**Total** ***env*** **positive**^**d**^	94/97 (97%)	10/41 (24%)
qPCR & RT-qPCR	Pol: P5	96/97 (99%)	21/41 (51%)

^a^Number positive/number tested (percent positive).

^b^37 and 5 positive samples in P3 PCR and RT-PCR, respectively, were not tested in P4.

^c^An animal was scored positive if it was PCR-positive on either of the pol primer pairs (P3 or P4).

^d^An animal was scored positive if it was PCR-positive on any of the four env primer pairs (P6, P7, P8 or P9).

Only 41 plasma RNA samples were available from SA koalas. In general, the level of positivity in conventional RT-PCRs was low, ranging from only 12% (5/41) for P2, P3, P8 and P9 RT-PCRs to 24% (10/41) for P6 RT-PCR (Table 3). On the RT-qPCR, 51% (21/41) of SA koalas were positive. When the RT-PCRs (including RT-qPCR) were grouped into genes, only 5/41 (12%) koalas were positive for all genes, 17/41 (42%) were positive for 1 or 2 genes, and 19/41 (46%) were negative for all genes. Complete results for each SA animal are presented in Supplementary Table 2.

### Completeness of KoRV provirus and gene transcription versus disease syndromes in SA koalas

The PCR status of proviral DNA of SA koalas (positive for all genes or not) exhibited a significant relationship with disease category (chi-square, P = 0.0014). As shown in Table 4, all koalas with neoplasia were positive for all proviral KoRV genes and were transcribing some or all genes, whereas no healthy or oxalate nephrosis animals were transcribing all viral genes. Animals with chlamydiosis or miscellaneous conditions fell in between these extremes.

**Table 4:** Proviral DNA and viral RNA gene positivity of SA koalas in each disease category. Some koalas appear in more than one disease category.

	Proviral DNA (total n = 97)			Viral RNA (total n = 41)		
	PCR-positive all genes^*^	PCR-positive in some but not all genes	PCR-negative all genes	PCR-positive all genes	PCR-positive in some but not all genes	PCR-negative all genes
Neoplasia (N = 5, DNA, 4, RNA)	5 (100%)	0 (0%)	0 (0%)	3 (75%)	1 (25%)	0 (0%)
Chlamydiosis (N = 34, DNA 17, RNA)	13 (38%)	21 (62%)	0 (0%)	2 (12%)	7 (41%)	8 (47%)
Oxalate nephrosis (N = 23, DNA, 9 RNA)	6 (26%)	16 (70%)	1 (4%)	0 (0%)	2 (22%)	7 (78%)
Miscellaneous (N = 15, DNA, 8 RNA)	10 (66%)	5 (33%)	0 (0%)	1 (13%)	4 (50%)	3 (38%)
Healthy (N = 26, DNA, N = 8 RNA)	6 (23%)	20 (77%)	0 (0%)	0 (0%)	3 (38%)	5 (62%)

^*^LTR is classed as a gene in this table.

### Proviral and Viral loads of QLD and SA koalas

**Figure 2:**
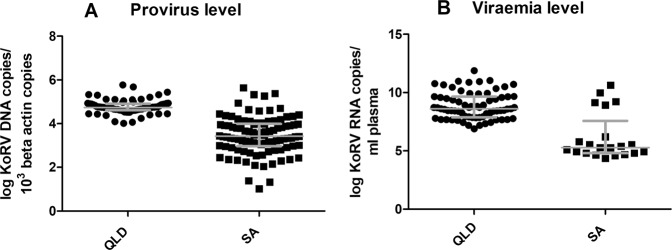
Comparison of (**A**) proviral DNA and (**B**) viral RNA loads between QLD (n = 71) and SA (DNA, n = 96; RNA, n = 21) koalas, evaluated using non-parametric Mann-Whitney U test. All resultant copy number values have been log_10_ transformed. Each dot denotes an individual’s KoRV load, Median line and interquartile range displayed. Y-axis shows individual koala values (log transformed) for (**A**) KoRV DNA copies/10^3^ *β-actin* copies and (**B**) KoRV RNA copies/ml plasma. X-axis is the location of the koala population. The proviral DNA and viral RNA load of KoRV was significantly (p < 0.0001) different between the populations.

The median proviral load in QLD koalas was 5.47 × 10^4^ KoRV DNA copies/10^3^ *β-actin* copies. The median proviral load of SA koalas was 2.71 × 10^3^ KoRV DNA copies/10^3^ *β-actin* copies, which was significantly lower than QLD koalas (two tailed Man Whitney U, P < 0.0001) (Fig. 2A).

Similarly, all QLD koalas had high plasma viral loads. The median viral load of QLD koalas was 4.07 × 10^8^ KoRV RNA copies/ml. Only 51.2% (21/41) SA koalas had detectable viral *pol* gene RNA and the median viral load was 1.89 × 10^5^ KoRV RNA copies/ml plasma. The viral load in SA koalas was significantly lower than those of QLD koalas (two tailed Man Whitney U, P < 0.0001) (Fig. 2B). Individual animal proviral and viral loads are presented in Supplementary Table 2.

**Figure 3:**
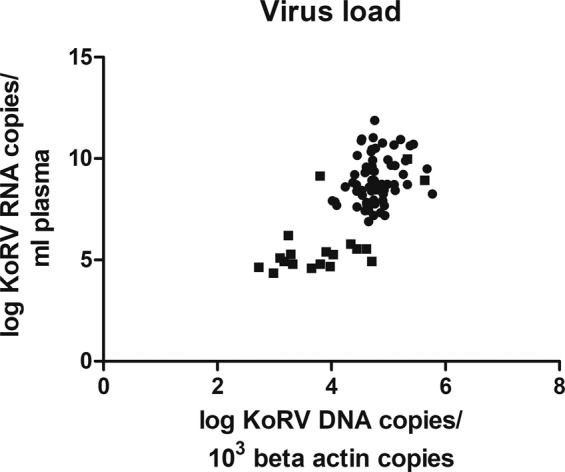
Correlation between *pol* gene proviral and viral loads of 91 QLD and SA koalas. X-axis shows individual koala values (log 10 transformed) of KoRV DNA copies/10^3^ *β-actin* copies; Y-axis shows (log 10 transformed) KoRV RNA copies/ml plasma of those koalas. SA animals are shown as squares and QLD animals as dots.

There was a significant correlation between proviral load and viral load (R^2^ = 0.4486, P value < 0.0001) across all animals, shown in Fig. 3.

**Figure 4:**
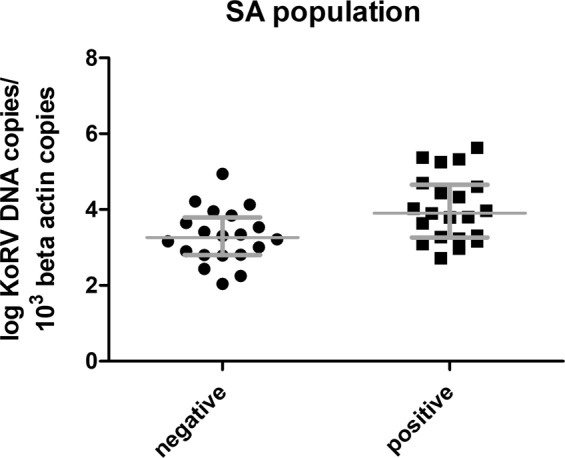
Comparison of KoRV *pol* proviral level between SA koalas that were RT-qPCR positive (20 animals) or negative (21 animals) for KoRV viral RNA in plasma. Median line and interquartile ranged displayed. Y-axis shows individual koala values (log transformed) of KoRV DNA copies/10^3^ *β-actin* copies.

The median proviral load was significantly higher (two tailed Mann Whitney-U, P value = 0.009) in SA animals that were actively transcribing virus (viral RNA positive) compared to koalas that were viral RNA negative (Fig. 4).

### Association between KoRV provirus and population characteristics

**Figure 5:**
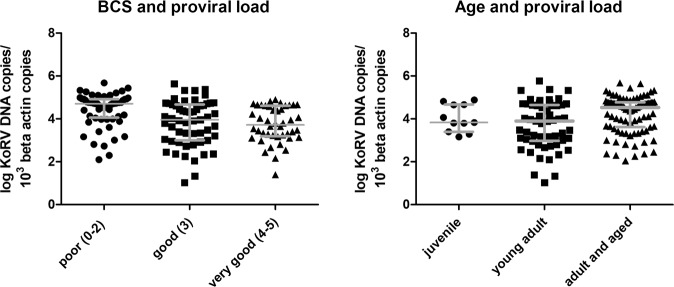
Comparison of KoRV *pol* proviral level and (**A**) body condition score (BCS) (61 poor, 55 good, 44 very good) and **B**) age (11 juvenile, 56 young adult and 90 adult and aged) of QLD and SA koalas. Y-axis shows individual koala values of log 10 KoRV DNA copies/10^3^ *β-actin* copies. Median line and interquartile range displayed.

As there were low numbers of SA koalas positive for viral RNA and viral load, statistical analysis of koala population characteristics and disease were performed with proviral loads only. No significant association was found between koalas’ sex (correlation) or body weight (Mann Whitney U) and proviral load. There was an association between lower body condition score and proviral load (Kruskal Wallis test, P < 0.0001, Dunns post hoc showed koalas with a “poor” body condition score had significantly higher median proviral loads than koalas with a “good” and “very good” body condition score (Fig. 5A). Similarly, there was an association between increasing proviral load and age (Kruskal Wallis P = 0.02, Dunns post hoc), with “adult and old” koalas having significantly higher median loads than “young adults” (Fig. 5B).

### Association of KoRV proviral loads with disease category

**Figure 6:**
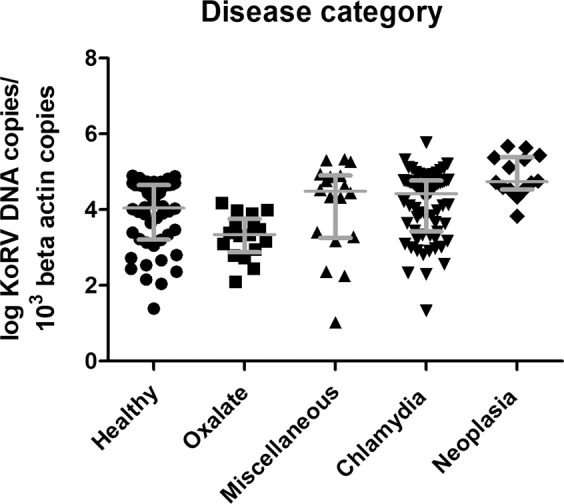
Comparison of KoRV *pol* proviral load and disease category of 48 healthy, 17 oxalate nephrosis, 18 miscellaneous, 68 chlamydiosis, 14 neoplasia QLD and SA koalas. Y-axis shows individual koala values of log 10 KoRV DNA copies/10^3^ β-actin copies. Median line and interquartile range displayed.

There were significant associations between median proviral load and some disease categories when each disease category was analysed against the median proviral load of the other categories combined (two tailed Mann Whitney U). Koalas with neoplasia (P = 0.009) and miscellaneous diseases (P = 0.0071) had significantly higher proviral loads, whereas animals with oxalate nephrosis (P < 0.0001) and healthy animals (P < 0.0001) had significantly lower proviral loads (Fig. 6). The proviral loads of animals with chlamydiosis were not significantly different to other disease categories.

### Multivariate modelling

Variables that showed a significant association with proviral load in the multivariate model were area of origin (QLD vs SA) and disease category (neoplasia), where samples from QLD had significantly higher proviral loads compared to samples from SA and samples that had neoplasia had significantly higher proviral loads compared to healthy samples. These effects were significant after controlling for effect of age, sex, body weight and body condition score of the koalas. The overall model explained 54% of total variability in proviral load in the study. Model results are presented in Table 5.

**Table 5:** Multivariate modelling outcomes, P values that were significant are in bold.

Variable Name		Coefficient	Standard Error	p-value	95% CI
Intercept		5.919	0.478		4.974 to 6.865
Area: (QLD V SA)		−1.173	0.136	**<0**.**001**	−1.442 to 0.904
Sex (F v M)		−0.177	0.114	0.124	−0.403 to 0.049
Body Weight		−0.0006	0.0019	0.746	−0.004 to 0.003
Age (Vs old)	adult	−0.1254584	0.1892652	0.508	−0.4995999 to 0.2486831
	young	−0.0713457	0.3335621	0.831	−0.730735 to 0.5880436
	juvenile	−0.015101	0.0653133	0.817	−0.1442131 to 0.1140111
Body condition score (Vs good)	Poor	0.1712522	0.149053	0.253	−0.1233973 to 0.4659017
	Very good	0.1385926	0.1494622	0.355	−0.156866 to 0.4340512
Disease Status (Vs healthy)	Chlamydiosis	0.0997492	0.1455689	0.494	−0.188013 to 0.3875114
	Neoplasia	0.6796565	0.2098902	**0**.**001**	0.2647431 to 1.09457
	Oxalate nephrosis	−0.1109544	0.2042469	0.588	−0.5147119 to 0.2928032
	Miscellaneous	0.1632565	0.1981873	0.411	−0.2285224 to 0.5550354

## Discussion

Koala populations are declining at a significant rate in most parts of Australia, particularly in Queensland, and koala retrovirus (KoRV) is likely to be contributing to the decline by inducing infectious diseases, such as chlamydiosis and neoplasia^19,21^. This study characterised the differences in extent of KoRV provirus (DNA) and viral expression (RNA) in QLD and SA koalas and investigated associations with disease.

All QLD koalas in the study were 100% positive for KoRV provirus *pol* DNA and viral *pol* RNA as has been reported in multiple studies^15,19^. In contrast, while 99% (n = 96/97) of SA koalas were positive for KoRV proviral DNA, only 51.2% (n = 21/41) had detectable viral RNA, based on qPCR and RT-qPCR of the *pol* gene, respectively. The high KoRV provirus prevalence of SA animals in the Mount Lofty Ranges was unexpected, as a recent study reported the provirus prevalence in wild-caught koalas in the same location to be 65% (49/75), detected by qPCR^23^. The same study and a previous study^14^ reported proviral prevalence of 42% and 15%, respectively, in wild-caught koalas on Kangaroo Island, SA. The higher proviral prevalence in SA koalas in the current study may have been influenced by the fact that the animals were rescued because of illness or injury, whereas the other studies were based on wild-caught animals that were predominantly healthy.

In line with data from the SA population generated by Illumina sequencing^16^ many SA animals did not test positive for all proviral gene segments of KoRV like their QLD counterparts. More interestingly even fewer of these animals had detectable RNA transcription for *gag*, *pol* or *env* genes. The absence of viral RNA in many of the proviral positive SA animals might be the result of a latent KoRV infection, where proviral DNA has been integrated into the host genome but gene transcription has been suppressed as occurs in feline leukemia virus infected cats^24–28^. It is also possible that these animals may not have full length viral loci present, resulting in inefficient viral transcription or they may have sequence variants of the virus that are not being detected by PCR assays. Full viral locus sequencing at DNA and RNA levels is required to resolve this, however this is not easy to achieve for retroviruses with the PCR based methods used in this study.

The variable prevalence of proviral genes and the absence of viral RNA in a large proportion of the SA koalas allows investigation of causal links between these KoRV parameters and disease, which is not possible in the 100% KoRV positive QLD koalas. In the SA animals, the presence of potentially full length provirus (all proviral genes) was associated with both transcription of viral RNA and disease category, where a positive association with neoplasia and a negative association with oxalate nephrosis (which is thought to be a genetic disorder unrelated to KoRV) were observed. The median KoRV proviral and viral loads in QLD koalas were significantly higher than those of SA koalas, which is in line with previous publications on proviral load^15,23^. In addition, a significant correlation between an individual’s proviral and viral loads were observed.

There are a number of potential explanations for the regional variation in viral loads between these two populations. Previous studies of genetic diversity have demonstrated genetic divergence between northern and southern koala populations^12,29,30^. The presence of the probable genetic disorder oxalate nephrosis solely in the SA population in this study also points strongly to genetic segregation between the QLD and SA animals^31^. It is possible that the genes encoding the known host receptors for KoRV infection (Pit1 and THTR1) are divergent between the two populations, analogous to the genetic resistance against murine leukaemia virus strains that occurs in some mouse strains^32^. Such differences in host receptor genes between QLD and SA koalas would potentially result in a reduced level of KoRV replication in the SA population. It is also possible that mutations in other host genes important in retroviral replication differ between the two populations, resulting in restricted KoRV replication in the SA animals.

Immune responses to the virus have been shown to vary between these populations in other studies^33,34^. QLD animals may become immunologically tolerant to the virus through *in-utero* expression of endogenous viral genes, resulting in an inability to respond effectively to infection by exogenous virus in later life, leading to high viral loads. However, studies investigating KoRV antibodies in QLD koalas have given mixed results, with some showing no detectable antibodies^35^ while others reported that Qld koalas in fact produced antibodies to multiple KoRV antigens^34^. In contrast, it has been suggested that SA animals might be infected only with an exogenous virus and as such are able to mount an immune response to the virus, as shown in a study in which both KoRV positive and negative koalas developed KoRV antibodies upon vaccination^34^.

Conversely, it is also possible that SA koalas might possess partial replication-defective endogenous KoRV sequences, which provide protection against exogenous virus replication. This is analogous to some endogenous Avian leucosis viruses^36^, Jaagsietke sheep retrovirus^37^ and Murine Leukaemia viruses^32^, in which endogenous *env* gene is expressed and defective envelope proteins are produced in some individuals. The env proteins effectively ‘blockade’ the retrovirus receptor, thereby preventing infection from exogenous viruses. This might explain the differences in proviral and viral loads between the two populations.

It was thought until recently that southern animals only possessed KoRV-A^14,23^ and not other variants of the virus and this might be a potential explanation for the reduced disease burden in southern animals. However our recent work has shown that South Australian animals do in fact have multiple variants of KoRV (including KoRV-B)^16,38^. The previous lack of detection of non-KoRV-A variants in southern populations is likely due to the use of relatively insensitive end point PCR for detection, together with the overall low proviral and viral loads in these populations.

The association between KoRV proviral loads and disease was explored at both a univariate and multivariate level. In univariate analysis koalas with a poor body condition score and older animals had higher proviral loads. The association between BCS and proviral load may be analogous to the wasting syndromes reported in association with other retroviruses such as HIV^39^. An association with age and viral load (though not a linear one) has been reported previously^19^ and this study partially confirms that. High KoRV proviral load was also associated with neoplasia, and “miscellaneous” diseases whereas healthy animals and those with the genetic disorder oxalate nephrosis had lower viral loads in univariate analysis. However when multivariate analysis was applied to control for multiple conditions and factors in individual animals the only parameters that remained firmly associated with proviral load were location of animal (with SA animals having lower proviral loads than QLD animals) and disease status (animals with neoplasia having higher proviral loads than healthy animals). The lack of a link between chlamydiosis and proviral load can partly be explained by the fact that most of the animals with neoplasia also had concurrent chlamydial disease. This study therefore supports earlier observations^19^ of a firm link between KoRV viral load and neoplasia but does not find a statistically significant association between proviral load and chlamydial disease as reported by^14^.

The link between KoRV loads and disease raises the issue of whether the association is causative or a consequence of disease. It is possible that the immunosuppression induced by chronic disease allows a subsequent escape of KoRV from immune control and increase in viral load and detection rates, i.e. that increased KoRV load is a consequence rather than a trigger of disease. However a recent longitudinal study of a large number of wild koalas^40^ indicates that KoRV viral load is relatively stable over time in an individual regardless of disease state and that high viral loads precede the development of clinical chlamydial disease rather than follow it.

This comparative population study provides some intriguing insights into KoRV and disease dynamics in these two genetically and geographically distinct koala populations. The QLD animals as reported in many studies are universally 100% positive for (presumably full length) KoRV with all gene segments detected. The SA animals on the other hand have lower KoRV proviral loads and not all genes are detectable in many animals. There is a strong link in both populations between proviral load and the viral load. The SA animals that did not possess all proviral genes were less likely to have detectable viral RNA and less likely to display KoRV associated disease. The underlying reasons for these population level differences in KoRV and disease are still not clear and require careful further work to untangle whether this is due to genetic variation in the animals (eg. the virus receptors), the presence or absence of endogenous versus exogenous forms of the virus (and therefore the animal’s ability to control viral replication immunologically) or blockade of KoRV infection by replication defective variants present only in the SA population.

## Supplementary information

Supplementary information

## References

[1] Canfield PJ, Sabine JM, Love DN (1988). Virus particles associated with leukaemia in a koala. Australian veterinary journal.

[2] Hanger JJ, Bromham LD, McKee JJ, O’Brien TM, Robinson WF (2000). The nucleotide sequence of koala (Phascolarctos cinereus) retrovirus: a novel type C endogenous virus related to Gibbon ape leukemia virus. Journal of virology.

[3] Tarlinton RE, Meers J, Young PR (2006). Retroviral invasion of the koala genome. Nature.

[4] Waugh CA (2017). Infection with koala retrovirus subgroup B (KoRV-B), but not KoRV-A, is associated with chlamydial disease in free-ranging koalas (Phascolarctos cinereus). Scientific reports.

[5] Tarlinton R, Meers J, Young P (2008). Biology and evolution of the endogenous koala retrovirus. Cellular and molecular life sciences: CMLS.

[6] Denner J, Young PR (2013). Koala retroviruses: characterization and impact on the life of koalas. Retrovirology.

[7] Canfield PJ, Brown AS, Kelly WR, Sutton RH (1987). Spontaneous lymphoid neoplasia in the koala (Phascolarctos cinereus). Journal of comparative pathology.

[8] Fabijan J (2017). Lymphoma, Koala Retrovirus Infection and Reproductive Chlamydiosis in a Koala (Phascolarctos cinereus). Journal of comparative pathology.

[9] Gonzalez-Astudillo V, Allavena R, McKinnon A, Larkin R, Henning J (2017). Decline causes of Koalas in South East Queensland, Australia: a 17-year retrospective study of mortality and morbidity. Scientific reports.

[10] Speight KN (2016). Prevalence and pathologic features of chlamydia pecorum infections in South Australian koalas (phascolarctos cinereus). Journal of wildlife diseases.

[11] Speight KN (2013). Pathological features of oxalate nephrosis in a population of koalas (Phascolarctos cinereus) in South Australia. Veterinary pathology.

[12] Neaves LE (2016). Phylogeography of the Koala, (Phascolarctos cinereus), and Harmonising Data to Inform Conservation. PloS one.

[13] Kjeldsen SR (2016). Genome-wide SNP loci reveal novel insights into koala (Phascolarctos cinereus) population variability across its range. Conservation Genetics.

[14] Legione AR (2017). Koala retrovirus genotyping analyses reveal a low prevalence of KoRV-A in Victorian koalas and an association with clinical disease. J Med Microbiol.

[15] Simmons GS (2012). Prevalence of koala retrovirus in geographically diverse populations in Australia. Australian veterinary journal.

[16] Tarlinton, R. E. *et al*. Differential and defective expression of Koala Retrovirus reveal complexity of host and virus evolution. *bioRxiv*, 211466, 10.1101/211466 (2017).

[17] Mellors JW (1996). Prognosis in HIV-1 Infection Predicted by the Quantity of Virus in Plasma. Science.

[18] Goto Y (2002). Association of plasma viral RNA load with prognosis in cats naturally infected with feline immunodeficiency virus. J Virol.

[19] Tarlinton R, Meers J, Hanger J, Young P (2005). Real-time reverse transcriptase PCR for the endogenous koala retrovirus reveals an association between plasma viral load and neoplastic disease in koalas. The Journal of general virology.

[20] Martin, R., Handasyde, K. A. & Simpson, S. *The koala: natural history*, *conservation and management*. (UNSW Press, 1999).

[21] Nyari S (2017). Epidemiology of chlamydial infection and disease in a free-ranging koala (Phascolarctos cinereus) population. PloS one.

[22] Sarker N (2018). Identification of stable reference genes for quantitative PCR in koalas. Scientific reports.

[23] Fabijan J (2019). Prevalence and clinical significance of koala retrovirus in two South Australian koala (Phascolarctos cinereus) populations. J Med Microbiol.

[24] Trono D (2010). HIV persistence and the prospect of long-term drug-free remissions for HIV-infected individuals. Science.

[25] Murphy B (2012). FIV establishes a latent infection in feline peripheral blood CD4+ T lymphocytes *in vivo* during the asymptomatic phase of infection. Retrovirology.

[26] Richman DD (2009). The challenge of finding a cure for HIV infection. Science.

[27] Dinoso JB (2009). A simian immunodeficiency virus-infected macaque model to study viral reservoirs that persist during highly active antiretroviral therapy. J Virol.

[28] Hartmann K (2012). Clinical Aspects of Feline Retroviruses: A Review. Viruses.

[29] Houlden BA, England PR, Taylor AC, Greville WD, Sherwin WB (1996). Low genetic variability of the koala Phascolarctos cinereus in south-eastern Australia following a severe population bottleneck. Molecular ecology.

[30] Ruiz-Rodriguez CT, Ishida Y, Greenwood AD, Roca AL (2014). Development of 14 microsatellite markers in the Queensland koala (Phascolarctos cinereus adustus) using next generation sequencing technology. Conservation genetics resources.

[31] Speight KN (2018). Necropsy findings of koalas from the Mount Lofty Ranges population in South Australia. Australian veterinary journal.

[32] Nethe M, Berkhout B, van der Kuyl AC (2005). Retroviral superinfection resistance. Retrovirology.

[33] Waugh C, Gillett A, Polkinghorne A, Timms P (2016). Serum Antibody Response to Koala Retrovirus Antigens Varies in Free-Ranging Koalas (Phascolarctos cinereus) in Australia: Implications for Vaccine Design. Journal of wildlife diseases.

[34] Olagoke O (2018). Induction of neutralizing antibody response against koala retrovirus (KoRV) and reduction in viral load in koalas following vaccination with recombinant KoRV envelope protein. NPJ vaccines.

[35] Fiebig U, Keller M, Moller A, Timms P, Denner J (2015). Lack of antiviral antibody response in koalas infected with koala retroviruses (KoRV). Virus research.

[36] Hunt H, Fadly A, Silva R, Zhang H (2008). Survey of endogenous virus and TVB* receptor status of commercial chicken stocks supplying specific-pathogen-free eggs. Avian Dis.

[37] Spencer TE, Mura M, Gray CA, Griebel PJ, Palmarini M (2003). Receptor usage and fetal expression of ovine endogenous betaretroviruses: implications for coevolution of endogenous and exogenous retroviruses. J Virol.

[38] Sarker N (2019). Genetic diversity of Koala retrovirus env gene subtypes: insights into northern and southern koala populations. J Gen Virol.

[39] Erlandson KM (2016). Long-term impact of HIV wasting on physical function. AIDS.

[40] Quigley, B. L. *et al*. Changes in Endogenous and Exogenous Koala Retrovirus Subtype Expression over Time Reflect Koala Health Outcomes. *Journal of virology* **93**, 10.1128/jvi.00849-19 (2019). 10.1128/JVI.00849-19 PMC6714790 31243137

[41] Miyazawa T, Shojima T, Yoshikawa R, Ohata T (2011). Isolation of koala retroviruses from koalas in Japan. The Journal of veterinary medical science.

