# Supplementary material for: Koala retrovirus viral load and disease burden in distinct northern and southern koala populations
Source: Sci Rep. 2020 Jan 14;10:263. doi: 10.1038/s41598-019-56546-0 (PMC6959342; doi:10.1038/s41598-019-56546-0)
Supplement: Supplementary file 1 — Supplementary information [file 41598_2019_56546_MOESM1_ESM.pdf]

Koala retrovirus viral load and disease burden in distinct northern and southern koala populations

Nishat Sarker[1,5], Jessica Fabijan[2], Helen Owen[1], Jennifer Seddon[1], Greg Simmons[1], Natasha Speight[2], Jasmeet Kaler[3], Lucy Woolford[2], Richard David Emes[3,4], Farhid Hemmatzadeh[2], Darren Trott[2], Joanne Meers[1*], and Rachael Tarlinton[3*]

[1]School of Veterinary Science, The University of Queensland, Australia
[2]School of Animal and Veterinary Sciences, The University of Adelaide, Australia
[3]School of Veterinary Medicine and Science, University of Nottingham, United Kingdom
[4]Advanced Data Analysis Centre (ADAC), University of Nottingham, United Kingdom
[5]Laboratory Sciences & Services Division, International Centre for Diarrhoeal Disease Research, Bangladesh

*Corresponding author:
Professor Joanne Meers and A/Prof Rachael Tarlinton
Email: Rachael.Tarlinton@nottingham.ac.uk

Table 1: Detailed information of studied koalas

study ID	Sex	Body Weight	Group	Tooth Wear	Body score
**UQ_QLD_01**	F	4.3	Adult	IV	2
**UQ_QLD_02**	F	3.3	Adult	IV	0
**UQ_QLD_03**	F	6.5	Adult	VI	1
**UQ_QLD_04**	M	6	Adult	IV	0
**UQ_QLD_05**	F	6.3	Old	VII	1
**UQ_QLD_06**	F	5.8	Young adult	II	0
**UQ_QLD_07**	M	4.5	Young adult	II	0
**UQ_QLD_08**	M	5.7	Young adult	II	0
**UQ_QLD_09**	M	7.6	Adult	IV	2
**UQ_QLD_10**	M	6.3	Adult	IV	2
**UQ_QLD_11**	M	7	Adult	IV	0
**UQ_QLD_12**	M	6.4	Adult	IV	2
**UQ_QLD_13**	F	5.5	Adult	V	3
**UQ_QLD_14**	M	7.7	Adult	V	2
**UQ_QLD_15**	F	4.8	Adult	IV	3
**UQ_QLD_16**	F	N/A	N/A	N/A	N/A
**UQ_QLD_17**	F	5	Adult	V	1
**UQ_QLD_18**	M	7.3	Adult	V	3
**UQ_QLD_19**	M	4.9	Adult	IV	0
**UQ_QLD_20**	M	5.1	Adult	IV	1
**UQ_QLD_21**	M	8.1	Adult	IV	1
**UQ_QLD_22**	M	7.7	Adult	V	1
**UQ_QLD_23**	M	6.8	Adult	IV	3
**UQ_QLD_24**	M	6.1	Adult	IV	0
**UQ_QLD_25**	F	5	Young adult	III	2
**UQ_QLD_26**	F	4.8	Adult	IV	1
**UQ_QLD_27**	M	5.5	Adult	IV	1
**UQ_QLD_28**	M	5.3	Adult	IV	2
**UQ_QLD_29**	F	4.7	Old	VII	2
**UQ_QLD_30**	M	8	Adult	IV	1
**UQ_QLD_31**	M	5.8	Adult	IV	1
**UQ_QLD_32**	M	6	Adult	IV	1
**UQ_QLD_33**	M	6.9	Adult	V	2
**UQ_QLD_34**	M	8.05	Adult	V	3
**UQ_QLD_35**	F	5	Young adult	III	2
**UQ_QLD_36**	M	6.05	Adult	V	2

**UQ_QLD_37**	F	5.6	Adult	V	2
**UQ_QLD_38**	M	7.81	Adult	VIII	3
**UQ_QLD_39**	M	8.5	Adult	VII	2
**UQ_QLD_40**	M	N/A	Adult	VIII	1
**UQ_QLD_41**	F	6	Adult	IV	3
**UQ_QLD_42**	F	5.3	Adult	VI	2
**UQ_QLD_43**	M	4.9	Young adult	II	2
**UQ_QLD_44**	F	5.7	Adult	VI	2
**UQ_QLD_45**	F	3.8	Old	VIII	1
**UQ_QLD_46**	F	4.9	Old	VII	1
**UQ_QLD_47**	M	6.6	Old	VII	2
**UQ_QLD_48**	M	6.5	Old	VIII	1
**UQ_QLD_49**	M	5.3	Young adult	III	N/A
**UQ_QLD_50**	M	4.9	Adult	V	3
**UQ_QLD_51**	F	5.23	Juvenile	I	4
**UQ_QLD_52**	F	5.73	Young adult	III	4
**UQ_QLD_53**	F	5.16	Adult	V	4
**UQ_QLD_54**	F	6.15	Adult	VI	3
**UQ_QLD_55**	F	5.58	Young adult	II	4
**UQ_QLD_56**	F	5.83	Young adult	II	4
**UQ_QLD_57**	F	5.16	Young adult	II	4
**UQ_QLD_58**	M	7.66	Young adult	II	4
**UQ_QLD_59**	M	6.6	Adult	V	3
**UQ_QLD_60**	M	5.93	Adult	IV	3
**UQ_QLD_61**	M	5.93	Young adult	II	3
**UQ_QLD_62**	M	8.59	Young adult	II	3
**UQ_QLD_63**	M	4.09	Young adult	II	4
**UQ_QLD_64**	F	4.11	Juvenile	I	4
**UQ_QLD_65**	M	4.54	Juvenile	I	4
**UQ_QLD_66**	F	3.97	Juvenile	I	3
**UQ_QLD_67**	F	3.61	Juvenile	I	4
**UQ_QLD_68**	F	5.05	Adult	V	3
**UQ_QLD_69**	F	7	Adult	VI	3
**UQ_QLD_70**	M	10.14	Adult	V	3
**UQ_QLD_71**	M	8.3	Adult	V	3
**UQ_SA_01**	M	10	Adult	IV	2
**UQ_SA_02**	M	9.3	young adult	III	4
**UQ_SA_03**	M	10.15	Adult	V	4
**UQ_SA_04**	F	5.4	young adult	II	2
**UQ_SA_05**	M	6.8	young adult	III	3

**UQ_SA_06**	F	N/A	Adult	IV	N/A
**UQ_SA_07**	M	9.2	N/A	N/A	3
**UQ_SA_08**	M	11	young adult	III	4
**UQ_SA_09**	M	9.15	Adult	IV	4
**UQ_SA_10**	M	8.5	Adult	IV	5
**UQ_SA_11**	F	3.85	juvenile	I	2
**UQ_SA_12**	F	7.05	young adult	III	2
**UQ_SA_13**	F	7.05	Adult	VI	2
**UQ_SA_14**	F	5.5	Adult	IV	2
**UQ_SA_15**	F	6.65	Adult	V	3
**UQ_SA_16**	F	6.76	young adult	II	3
**UQ_SA_17**	M	11.35	Adult	IV	3
**UQ_SA_18**	M	6.7	juvenile	I	5
**UQ_SA_19**	F	6.5	young adult	III	2
**UQ_SA_20**	F	8.15	young adult	III	3
**UQ_SA_21**	F	N/A	young adult	II	2
**UQ_SA_22**	M	9.35	Adult	IV	3
**UQ_SA_23**	F	N/A	Adult	IV	4
**UQ_SA_24**	M	8.06	Adult	V	3
**UQ_SA_25**	M	N/A	Adult	V	3
**UQ_SA_26**	M	8.9	Adult	VI	4
**UQ_SA_27**	F	N/A	Adult	IV	2
**UQ_SA_28**	M	9.9	Adult	IV	2
**UQ_SA_29**	M	9.95	young adult	III	5
**UQ_SA_30**	F	4.75	Adult	IV	4
**UQ_SA_31**	M	2.8	juvenile	I	3
**UQ_SA_32**	F	7.25	Adult	VI	3
**UQ_SA_33**	F	4.7	young adult	II	3
**UQ_SA_34**	F	5.3	young adult	III	3

**UQ_SA_35**	M	10.7	Adult	IV	4
**UQ_SA_36**	M	7	young adult	II	3
**UQ_SA_37**	F	N/A	N/A	N/A	3
**UQ_SA_38**	M	N/A	Adult	IV	5
**UQ_SA_39**	M	10	young adult	III	5
**UQ_SA_40**	F	6.1	young adult	III	5
**UQ_SA_41**	M	9.3	Adult	IV	2
**UQ_SA_42**	M	5.2	young adult	II	5
**UQ_SA_43**	M	N/A	young adult	II	5
**UQ_SA_44**	N/A	N/A	young adult	II	5
**UQ_SA_45**	M	N/A	young adult	II	5
**UQ_SA_46**	M	N/A	Adult	IV	4
**UQ_SA_47**	F	7.3	young adult	III	N/A
**UQ_SA_48**	M	N/A	N/A	N/A	N/A
**UQ_SA_49**	F	N/A	N/A	N/A	N/A
**UQ_SA_50**	F	N/A	Adult	IV	2
**UQ_SA_51**	M	2.26	juvenile	I	5
**UQ_SA_52**	M	11.8	Adult	IV	4
**UQ_SA_53**	M	N/A	young adult	II	3
**UQ_SA_54**	F	5.6	young adult	III	3
**UQ_SA_55**	F	0.8	juvenile	I	3
**UQ_SA_56**	F	6.43	Adult	IV	3
**UQ_SA_57**	M	8	Adult	IV	3
**UQ_SA_58**	F	3.6	juvenile	I	5
**UQ_SA_59**	F	7.2	Adult	IV	4
**UQ_SA_60**	M	8.6	Adult	IV	2
**UQ_SA_61**	F	N/A	N/A	N/A	3
**UQ_SA_62**	F	N/A	Adult	IV	3
**UQ_SA_63**	M	N/A	Adult	VI	3
**UQ_SA_64**	M	3.8	juvenile	I	3
**UQ_SA_65**	M	7.27	young adult	III	2
**UQ_SA_66**	F	4.46	young adult	III	2
**UQ_SA_67**	F	5.75	young adult	III	4
**UQ_SA_68**	M	10.8	young adult	III	5
**UQ_SA_69**	M	N/A	young adult	II	3
**UQ_SA_70**	F	7.6	Adult	IV	4
**UQ_SA_71**	M	10.74	young adult	III	5
**UQ_SA_72**	F	8	young adult	III	2
**UQ_SA_73**	F	5.5	young adult	II	2
**UQ_SA_74**	M	8.7	young adult	III	2
**UQ_SA_75**	M	9.2	young adult	III	4
**UQ_SA_76**	M	7.72	young adult	III	2
**UQ_SA_77**	M	8.2	young adult	III	3
**UQ_SA_78**	F	6.5	Adult	IV	3

**UQ_SA_79**	M	11	N/A	N/A	4
**UQ_SA_80**	M	10.5	Adult	V	4
**UQ_SA_81**	M	8	Adult	IV	1
**UQ_SA_82**	M	7.4	Adult	IV	N/A
**UQ_SA_83**	M	7	young adult	II	3
**UQ_SA_84**	F	6.12	young adult	III	3
**UQ_SA_85**	M	8.52	young adult	III	4
**UQ_SA_86**	M	9.3	Adult	IV	3
**UQ_SA_87**	F	9.8	Adult	IV	3
**UQ_SA_88**	M	7.73	Adult	V	3
**UQ_SA_89**	M	6.8	young adult	II	4
**UQ_SA_90**	M	11.75	Adult	IV	3
**UQ_SA_91**	M	9	young adult	III	3
**UQ_SA_92**	F	6.03	young adult	III	2
**UQ_SA_93**	F	6.97	young adult	III	3
**UQ_SA_94**	M	11.2	young adult	III	5
**UQ_SA_95**	F	9.7	Adult	IV	3
**UQ_SA_96**	F	6.6	Adult	IV	3
**UQ_SA_97**	M	9.9	Adult	IV	3
**UQ_SA_98**	M	5.95	young adult	II	3
**UQ_SA_99**	M	8.15	young adult	III	5
**UQ_SA_100**	F	9	Adult	IV	4
**UQ_SA_101**	M	10.35	young adult	III	5
**UQ_SA_102**	F	8	Adult	VI	3
**UQ_SA_103**	M	11.7	Adult	VI	3
**UQ_SA_104**	M	10.65	Adult	IV	3
**UQ_SA_105**	F	7.55	Adult	IV	2

*UQ_QLD is QLD koalas and UQ_SA is SA koalas

* N/A = sample not available to test

**Pathology**	**Clinical status**
Chronic cystitis, interstitial nephritis	Chlamydiosis
Moderate Chronic Cystitis and emaciation	Chlamydiosis
Bilateral conjuntivitis, mild chronic meningitis, focal liver neoplasia, focal granulomatous lymphadenitis	Chlamydiosis
myocardial fibrosis	Miscellaneous
Bilateral conjuntivitis	Chlamydiosis
Possible acute pulmonary oedema. Severe respiratory noises	Miscellaneous
focal aspiration pneumonia, myocardial fibrosis, Mild to moderate lymphoplasmacytic cystitis	Chlamydiosis
Multisystem lymphoma, Anaemia, Periacinar coagulative necrosis	Neoplasia
Grade 3 bilateral conjunctivitis. Grade 1 cystitis	Chlamydiosis
grade 3 Bilateral conjunctivitis and keratitis. Intestinal cestodes.	Chlamydiosis
Pulmonary fibrosis, atelectasis and emphysema	Miscellaneous
Grade 3 Bilateral conjunctivitis, Multifocal to coalescing, moderate, neutrophilic myocarditis, locally extensive pancreatic fibrosis	Chlamydiosis
Cystitis, bilateral paraovarian cysts	Chlamydiosis
Bilateral conjunctivitis; Grade2, focal splenic fibosis, Hepatic nodular hyperplasia, Intestinal helminthiasis.	Chlamydiosis
bilateral paratubular ovarian cysts	Chlamydiosis
Non-neoplastic hepatic mass	Miscellaneous
Grade 3 bilateral conjunctivitis. Bilateral severe paraovarian cysts. Heart: multifocal, mild myocardial fibrosis. Lungs: multifocal interstitial fibrosis, mild neutrophilic alveolitis	Chlamydiosis
Grade 3 bilateral conjunctivitis.	Chlamydiosis
Grade 3 Cystitis. Heart: minimal, multifocal fibrosis; Adrenal gland: locally extensive medullary fibrosis; Emaciation and serous atrophy of adipose tissue.	Chlamydiosis
Conjunctivitis and Grade 3 Cystitis. Left kidney: severe pyogranulomatous nephritis; Brain: mild choroiditis	Chlamydiosis
Mesothelioma	Neoplasia
Bilateral conjunctivitis grade 3. Mild, chronic cystitis. Mild, multifocal, neutrophilic and granulomatous pneumonia. Mild, multifocal myocardial fibrosis. Serous atrophy. Testicular tubular degeneration. Right hip joint mild osteoarthritis.	Chlamydiosis
Bilateral Conjunctivitis. Multifocal, ulcerative stomatitis associatd with teeth	Chlamydiosis
Bilateral Conjunctivitis. Emaciation. Kidney: locally extensive interstitial fibrosis, equivocal glomerulonephrosis.	Chlamydiosis
Cystitis, vaginitis, dermatitis over left mammary gland	Chlamydiosis
Lung: mild interstitial pneumonia and multifocal macrophages, possibly with cytoplasmic lipid in alveoli. Bilateral paraovarian cysts. Skull: osteochondroma	Neoplasia , Chlamydiosis
lymphoid leukaemia	Neoplasia
Bilateral Conjunctivitis, Cystitis. Osteochondroma	Neoplasia , Chlamydiosis
Bilateral Conjunctivitis, Grade 4 Cystitis.	Chlamydiosis
Wasted, temporal muscle degeneration and regeneration	Miscellaneous
Multiple fractures, mild cystitis, mild neutrophilic and necrotising hepatitis	Chlamydiosis
Cystitis	Chlamydiosis
lymphoid leukaemia, lymphocytosis	Neoplasia
Urogenital Chlamydia, craniofacial tumour, ear Infection	Neoplasia , Chlamydiosis
old bruising, collapsed lung, luxated shoulder, fresh pleural bleeding. Emaciated.	Miscellaneous
Urogenital Chlamydiosis, leukaemia, severe anaemia	Neoplasia , Chlamydiosis

Lymphoma, Chlamydial conjunctivitis, ringworm, leucopenia	Neoplasia , Chlamydiosis
Bilateral Conjuntivitis and cystitis, wasting, early peritonitis	Chlamydiosis
Conjunctivitis, Worsening Lymphocytosis	Chlamydiosis
Mild Conjuntivitis-right eye, Cystitis	Chlamydiosis
Cystitis	Chlamydiosis
Chlamydial Conjunctivitis	Chlamydiosis
Conjunctivitis, Chronic nephritis.<br>Heart: Hypertrophy and fibrosis of left ventricle.	Chlamydiosis
lymphoplasmacytic cystitis.<br>Likely oesophageal candidasis.<br>Mild myocardial fibrosis.	Chlamydiosis
Mild Conjuntivitis-left eye, cystitis	Chlamydiosis
low BCS	Miscellaneous
Moderate Conjuntivitis- left eye, hyperkeratosis, fungal infection with scabs	Chlamydiosis
Wasted	Miscellaneous
Cystitis, Bone marrow aplasia	Chlamydiosis
Not Required	Healthy
Not Required	Healthy
Not Required	Healthy
Not Required	Healthy
Clinically Healthy and live captive	Healthy
Not Required	Healthy
Not Required	Healthy
Not Required	Healthy
Not Required	Healthy
Not Required	Healthy
Not Required	Healthy
Not Required	Healthy
Not Required	Healthy
Not Required	Healthy
Not Required	Healthy
Not Required	Healthy
Not Required	Healthy
Not Required	Healthy
Not Required	Healthy
Not Required	Healthy
Not Required	Healthy
Not Required	Healthy
No abnormalities detected	Miscellaneous
Sarcoptic mange	Miscellaneous
Pharyngeal impaction; aspiration pneumonia	Miscellaneous
Oxalate nephrosis	Oxalate nephrosis
Lymphosarcoma (LN -cervical, auxillary, submandibular, spleen)	Neoplasia

Mange, oxalate nephrosis, cystitis, paraovarian cysts	Chlamydiosis, oxalate nephrosis, Miscellaneous
Severe seizures, No abnormalities detected	Miscellaneous
Urogenital chlamydiosis,<br>Prostate: chronic active mixed periurethral prostatitis<br>Bladder: mild cystic haemorrhage	Chlamydiosis
Oxalate nephrosis	Oxalate nephrosis
Oxalate nephrosis	Oxalate nephrosis
Oxalate nephrosis	Oxalate nephrosis
Oxalate nephrosis; paraovarian cyst, endometriosis	Chlamydiosis, oxalate nephrosis
subcutaneous oedema (shoulder and dorsal head/ neck region); paraovarian cyst; Kidneys: chronic cortical loss and fibrosis.	Chlamydiosis
Gastrointestinal issues	Miscellaneous
Multicentric lymphosarcoma (spleen, adrenal gland, pancreas, mesenteric lymph nodes, ovary, bone marrow); pyometra; right femoral condyle: severe erosive arthritis with marrow lymphosarcoma	Neoplasia, Chlamydiosis
Lymphosarcoma	Neoplasia
Urethral and prostate inflammation, tooth abscess, mild conjunctivitis	Chlamydiosis
dog attack	Healthy
Non-chlamydial endometritis, Pyometra, dental disease, arthritis	Chlamydiosis
Mange	Miscellaneous
Oxalate nephrosis; severe cystitis	Chlamydiosis, oxalate nephrosis
Chlamydiosis -<br>Prostate: moderate to severe chronic-active mixed prostatitis<br>Kidney: chronic lymphoplasmacytic (ascending) pyelonephritis; segmental tubular loss, fibrosis and regeneration<br>Bladder: chronic-active mixed ulcerative haemorrhagiv cystitis	Chlamydiosis
Chlamydiosis -<br>Cyst: necrosuppurative and granulomatous ovarian bursitis<br>Uterus: very mild suppurative endometritis	Chlamydiosis
Likely septic infection from mandible fracture -<br>Nephritis, lymphadentitis<br>Kidney: focal severe cotricomedullary fibrosuppurative and necrotising nephritis (embolic) with intralesional bacteria. The wedge shaped appearance of the lesion is typical or embolic/haematogenous infection likely secondary to osteomylitis<br>Mild pyogranulomatous lymphadenitis	Miscellaneous
Infection from an unknown trauma	Healthy
Aspiratory pneumonia	Miscellaneous
Oxalate nephrosis, non-chlamydial endometriosis	Oxalate nephrosis
non-chlamydial reproductive clinical disease	Miscellaneous
Reproductive changes likely due to trauma/infarction - non-infectious	Healthy
Endometritis	Chlamydiosis
No chronic abnormalities detected by histology - unwell	Miscellaneous
Mild cystitis	Chlamydiosis
Oxalate nephrosis	Oxalate nephrosis
Oxalate nephrosis	Chlamydiosis, Oxalate nephrosis

Cystitis, dog attack (inguinal, hindlimb, thoracic and abdominal wounds)	Chlamydiosis
Mandibular osteomyelitis	Healthy
thromboembolic disease	Miscellaneous
Prostatitis and urethritis, dog attack (abdominal, rump and facial wounds)	Chlamydiosis
Vehicle trauma	Healthy
No abnormalities detected	Miscellaneous
Urinary chlamydiosis	Chlamydiosis
Genetic disorder - scoliosis and kyphosis	Healthy
Chlamydiosis - conjunctivitis, cystitis	Chlamydiosis
HBC (traumatic injuries to the mandible and head)	Healthy
Oxalate nephrosis	Oxalate nephrosis
Prostatitis	Chlamydiosis
Conjunctivitis, trumatic injuries	Chlamydiosis
Insufficient information	N/A
Dog related trauma	Healthy
Oxalate nephrosis	Oxalate nephrosis
Twisted bowel	Miscellaneous
Conjunctivitis, urethritis	Chlamydiosis
Chlamydiosis - urethritis, prostatitis; gastrointestinal perforation	Chlamydiosis
Oxalate nephrosis	Oxalate nephrosis
Cystitis, gastrointestinal issues	Chlamydiosis
Oxalate nephrosis	Oxalate nephrosis
Insufficient information	N/A
Scoliosis and kyphosis, pneumonia	Chlamydiosis
Endometriosis, paraovarian cysts	Chlamydiosis
Oxalate nephrosis, prostatitis	Chlamydiosis, Oxalate nephrosis
Craniofacial tumour	Neoplasia
Endometritis, conjunctivitis	Chlamydiosis
Genetic disorder - scoliosis and kyphosis	Healthy
Lymphosarcoma	Neoplasia
Oxalate nephrosis	Oxalate nephrosis
Vehicle trauma	Healthy
Vehicle trauma	Healthy
Vehicle trauma	Healthy
Oxalate nephrosis	Oxalate nephrosis
Dog related trauma	Healthy
Vehicle trauma	Healthy
Dog related trauma	Healthy
Oxalate nephrosis	Oxalate nephrosis
Vehicle trauma, oxalate nephrosis	Oxalate nephrosis
Vehicle trauma	Healthy
Nephritis, cystitis, prostatitis, urethritis	Chlamydiosis
Vehicle trauma	Healthy
Nephritis, Dog attack	Chlamydiosis

Trauma	Healthy
Cystitis, prostatitis, urethritis , dog attack	Chlamydiosis
Oxalate nephrosis	Oxalate nephrosis
Scoliosis and kyphosis, Nephritis, pyelonephritis, urethritis, prostatitis, cystitis	Chlamydiosis
Mange	Miscellaneous
Oxalate nephrosis	Oxalate nephrosis
Dog related trauma	Healthy
Pyelonephritis, cystitis, urethritis	Chlamydiosis
No abnormalities detected	Healthy
keratitis, paraovarian cysts, vaginitis	Chlamydiosis
No abnormalities detected	Healthy
Dog attack, Mild cystitis and urethritis, small testicle	Chlamydiosis
No abnormalities detected by histology - unwell	Miscellaneous
Endometritis	Chlamydiosis
Hit by car, Endometritis and cystitis	Chlamydiosis
Vehicle trauma	Healthy
Vehicle trauma	Healthy
Hit by car, Mild cystitis, paraovarian cysts	Chlamydiosis
Trauma causing infection, cystitis	Miscellaneous
Oxalate nephrosis	Oxalate nephrosis
Vehicle trauma	Healthy
Trauma, Paraovarian cysts, kidney disease	Chlamydiosis
Trauma	Healthy
Cystitis and paraovarian cysts	Chlamydiosis
Trauma - broken spine	Healthy
Oxalate nephrosis, prostatitis	Oxalate nephrosis
Chlamydiosis - endometritis, paraovarian cysts	Chlamydiosis

**Overall Chlamydia status**	**Chlamydiosis status**	***Chlamydia pecorum* status**	***Chlamydia peocrum* DNA copies/ul**
Overt chlamydial disease - PCR negative	urogenital	negative	N/A
Overt chlamydial disease - PCR positive	urogenital	urogenital	1.91E+04
Overt chlamydial disease - PCR negative	ocular	negative	N/A
Chlamydia negative	negative	negative	N/A
Overt chlamydial disease - PCR positive	ocular	ocular	4.55E+02
Chlamydia negative	negative	negative	N/A
Overt chlamydial disease - PCR negative	urogenital	negative	N/A
Chlamydia negative	negative	negative	N/A
Overt chlamydial disease - PCR negative	ocular, urogenital	negative	N/A
Overt chlamydial disease - PCR negative	ocular	negative	N/A
Chlamydia negative	negative	negative	N/A
Overt chlamydial disease - PCR negative	ocular	negative	N/A
Overt chlamydial disease - PCR positive	urogenital	urogenital	1.50E+03
Overt chlamydial disease - PCR negative	ocular	negative	N/A
Overt chlamydial disease - PCR positive	urogenital	urogenital	9.51E+02
Chlamydia negative	negative	negative	N/A
Overt chlamydial disease - PCR positive	ocular, urogenital	ocular	1.41E+03
Overt chlamydial disease - PCR negative	ocular	negative	N/A
Overt chlamydial disease - PCR negative	urogenital	negative	N/A
Overt chlamydial disease - PCR negative	ocular, urogenital	negative	N/A
Chlamydia negative	negative	negative	N/A
Overt chlamydial disease - PCR negative	ocular, urogenital	negative	N/A
Overt chlamydial disease - PCR positive	ocular	ocular	7.96E+02
Overt chlamydial diseasel disease - PCR negative	ocular	negative	N/A
Overt chlamydial disease - PCR negative	urogenital	negative	N/A
Overt chlamydial disease - PCR negative	urogenital	negative	N/A
Chlamydia negative	negative	negative	N/A
Overt chlamydial disease - PCR positive	ocular, urogenital	ocular	1.62E+02
Overt chlamydial disease - PCR negative	ocular, urogenital	negative	N/A
Chlamydia negative	negative	negative	N/A
Overt chlamydial disease - PCR positive	urogenital	urogenital	2.90E+02
Overt chlamydial disease - PCR negative	urogenital	negative	N/A
Chlamydia negative	negative	N/A	N/A
Overt chlamydial disease - PCR positive	urogenital	urogenital	1.01E+06
Chlamydia negative	negative	negative	N/A
Overt chlamydial disease - PCR positive	urogenital	urogenital	2.59E+04

Overt chlamydial disease - PCR negative	ocular	negative	N/A
Overt chlamydial disease - PCR negative	ocular, urogenital	negative	N/A
Overt chlamydial disease - PCR positive	ocular	ocular	1.20E+02
N/A	ocular, urogenital	N/A	N/A
N/A	urogenital	N/A	N/A
N/A	ocular	N/A	N/A
Overt chlamydial disease - PCR positive	ocular	ocular	309 (LE)
Overt chlamydial disease - PCR positive	ocular	ocular, urogenital	32942 (RE) + 793 (U)
N/A	ocular, urogenital	N/A	N/A
N/A	negative	N/A	N/A
N/A	N/A	N/A	N/A
N/A	negative	N/A	N/A
N/A	urogenital	N/A	N/A
N/A	negative	N/A	N/A
Chlamydia negative	negative	negative	N/A
Chlamydia negative	negative	negative	N/A
Chlamydia negative	negative	negative	N/A
Subclinical	negative	ocular	231 (RE)
Chlamydia negative	negative	negative	N/A
Chlamydia negative	negative	negative	N/A
Chlamydia negative	negative	negative	N/A
Chlamydia negative	negative	negative	N/A
Chlamydia negative	negative	negative	N/A
Chlamydia negative	negative	negative	N/A
Chlamydia negative	negative	negative	N/A
Chlamydia negative	negative	negative	N/A
Chlamydia negative	negative	negative	N/A
Chlamydia negative	negative	negative	N/A
Chlamydia negative	negative	negative	N/A
Chlamydia negative	negative	negative	N/A
Chlamydia negative	negative	negative	N/A
Chlamydia negative	negative	negative	N/A
Chlamydia negative	negative	negative	N/A
Chlamydia negative	negative	negative	N/A
Chlamydia negative	negative	negative	N/A
Chlamydia negative	negative	negative	N/A
Chlamydia negative	negative	negative	N/A
Chlamydia negative	negative	negative	N/A
Subclinical chlamydia	negative	urogenital	2.30E+02
Chlamydia negative	negative	negative	N/A

Overt chlamydial disease - PCR positive	urogenital	urogenital	1.12E+03
Subclinical	negative	urogenital	1.80E+02
Overt chlamydial disease - PCR positive	urogenital	ocular	9.10E+01
Chlamydia negative	negative	negative	N/A
Chlamydia negative	negative	negative	N/A
Subclinical	negative	ocular	1.68E+02
Overt chlamydial disease - PCR positive	urogenital	urogenital	1.66E+03
Overt chlamydial disease - PCR negative	urogenital	negative	N/A
Chlamydia negative	negative	negative	N/A
Overt chlamydial disease - PCR positive	urogenital	urogenital	2.00E+01
Chlamydia negative	negative	negative	N/A
Overt chlamydial disease - PCR positive	ocular, urogenital	ocular, urogenital	30, 104
Chlamydia negative	negative	negative	N/A
Overt chlamydial disease - PCR negative	urogenital	negative	N/A
N/A	N/A	N/A	N/A
Overt chlamydial disease - PCR positive	urogenital	urogenital	N/A
Overt chlamydial disease - PCR positive	urogenital	urogenital	N/A
Overt chlamydial disease - PCR positive	urogenital	urogenital	N/A
Chlamydia negative	N/A	negative	N/A
Subclinical	negative	urogenital	1.94E+04
N/A	N/A	N/A	N/A
N/A	N/A	N/A	N/A
N/A	N/A	N/A	N/A
Chlamydia negative	negative	negative	N/A
Overt chlamydial disease - PCR positive	urogenital	ocular, urogenital	LE 3656 and RE 11281, 12410
Chlamydia negative	negative	negative	N/A
Overt chlamydial disease - PCR negative	urogenital	negative	N/A
Chlamydia negative	negative	negative	N/A
Overt chlamydial disease - PCR positive	urogenital	urogenital	3.73E+04

Overt chlamydial disease - PCR positive	urogenital	ocular, urogenital	4197, 4424
Chlamydia negative	negative	negative	N/A
N/A	N/A	N/A	N/A
Overt chlamydial disease - PCR negative	urogenital	negative	N/A
N/A	N/A	N/A	N/A
Chlamydia negative	negative	negative	N/A
Overt chlamydial disease - PCR positive	urogenital	ocular, urogenital	100, 200
Chlamydia negative	negative	negative	N/A
Overt chlamydial disease - PCR positive	ocular, urogenital	ocular, urogenital	30, 35
Chlamydia negative	negative	negative	N/A
N/A	N/A	N/A	N/A
Chlamydia negative	negative	negative	N/A
Overt chlamydial disease - PCR positive	ocular	pooled	8.39E+02
N/A	N/A	N/A	N/A
N/A	N/A	N/A	N/A
Chlamydia negative	negative	negative	N/A
Subclinical	negative	ocular, urogenital	N/A
Chlamydia negative	negative	negative	N/A
Overt chlamydial disease - PCR positive	urogenital	ocular, urogenital	500, 150
Subclinical	negative	urogenital	1.44E+03
N/A	N/A	N/A	N/A
Chlamydia negative	negative	negative	N/A
N/A	N/A	N/A	N/A
Overt chlamydial disease - PCR positive	ocular, urogenital	ocular, urogenital	1500, 1000
N/A	urogenital	N/A	N/A
N/A	negative	N/A	N/A
N/A	negative	N/A	N/A
N/A	ocular	N/A	N/A
N/A	negative	N/A	N/A
Chlamydia negative	negative	negative	N/A
Chlamydia negative	negative	negative	N/A
Chlamydia negative	negative	negative	N/A
Chlamydia negative	negative	negative	N/A
Subclinical	negative	urogenital	1.65E+03
N/A	negative	N/A	N/A
Subclinical	negative	ocular, urogenital	513, 1800
Chlamydia negative	negative	negative	N/A
Chlamydia negative	negative	negative	N/A
Chlamydia negative	negative	negative	N/A
Chlamydia negative	negative	negative	N/A
Chlamydia negative	negative	negative	N/A
Overt chlamydial disease - PCR negative	urogenital	negative	N/A
Chlamydia negative	negative	negative	N/A
N/A	negative	N/A	N/A

N/A	negative	N/A	N/A
Overt chlamydial disease - PCR negative	urogenital	negative	N/A
Chlamydia negative	negative	negative	N/A
Overt chlamydial disease - PCR negative	urogenital	negative	N/A
Chlamydia negative	negative	negative	N/A
Chlamydia negative	negative	negative	N/A
Chlamydia negative	negative	negative	N/A
Overt chlamydial disease - PCR negative	urogenital	negative	N/A
Chlamydia negative	negative	negative	N/A
Overt chlamydial disease - PCR negative	urogenital	negative	N/A
Subclinical chlamydia	negative	ocular	8.11E+02
Overt chlamydial disease - PCR negative	urogenital	negative	N/A
Chlamydia negative	negative	negative	N/A
Overt chlamydial disease - PCR positive	urogenital	urogenital	N/A
Overt chlamydial disease - PCR negative	urogenital	negative	N/A
Subclinical	negative	urogenital	4.22E+03
Chlamydia negative	negative	negative	N/A
Overt chlamydial disease - PCR positive	urogenital	urogenital	5.43E+02
Chlamydia negative	negative	negative	N/A
Chlamydia negative	negative	negative	N/A
Chlamydia negative	negative	negative	N/A
Overt chlamydial disease - PCR negative	urogenital	negative	N/A
Chlamydia negative	negative	negative	N/A
Overt chlamydial disease - PCR negative	urogenital	negative	N/A
Chlamydia negative	negative	negative	N/A
Chlamydia negative	negative	negative	N/A
Overt chlamydial disease - PCR positive	urogenital	urogenital	3.20E+06

Table 2: PCR results of SA koalas

	DNA						
study ID	pol			any pol (real time+conventional)	LTR	Gag	
	P3	P4	provirus load/ 1000 B-actin copies based on pol qPCR				P8
UQ_SA_01	pos	pos	2.55E+03	pos	pos	pos	pos
UQ_SA_02	neg	pos	1.25E+03	pos	pos	pos	pos
UQ_SA_03	pos	pos	2.78E+04	pos	pos	pos	pos
UQ_SA_04	pos	pos	1.53E+04	pos	pos	neg	neg
UQ_SA_05	pos	pos	2.14E+05	pos	pos	pos	pos
UQ_SA_06	neg	neg	2.63E+03	pos	neg	neg	neg
UQ_SA_07	pos	pos	2.16E+04	pos	pos	pos	pos
UQ_SA_09	pos	pos	2.03E+03	pos	pos	pos	pos
UQ_SA_10	neg	pos	3.45E+03	pos	pos	pos	pos
UQ_SA_11	neg	pos	1.45E+03	pos	pos	neg	neg
UQ_SA_12	neg	neg	1.35E+04	pos	neg	neg	neg
UQ_SA_13	neg	pos	6.42E+02	pos	pos	pos	neg
UQ_SA_14	pos	pos	1.82E+05	pos	pos	pos	pos
UQ_SA_15	pos	pos	4.32E+05	pos	pos	pos	pos
UQ_SA_16	pos	pos	2.39E+05	pos	pos	pos	pos
UQ_SA_17	neg	pos	2.07E+03	pos	pos	pos	neg
UQ_SA_18	neg	pos	2.51E+03	pos	neg	neg	neg
UQ_SA_19	neg	pos	1.47E+03	pos	neg	neg	neg
UQ_SA_21	neg	pos	1.03E+03	pos	pos	neg	neg
UQ_SA_22	pos	pos	4.08E+04	pos	pos	pos	pos
UQ_SA_23	pos	pos	3.84E+04	pos	pos	pos	neg
UQ_SA_24	neg	pos	1.78E+02	pos	pos	pos	neg
UQ_SA_25	pos	pos	2.16E+03	pos	pos	pos	neg
UQ_SA_27	neg	pos	4.02E+03	pos	pos	pos	neg
UQ_SA_28	neg	pos	1.29E+04	pos	pos	pos	pos
UQ_SA_29	neg	pos	1.67E+03	pos	pos	pos	pos
UQ_SA_30	neg	pos	2.53E+04	pos	pos	pos	pos
UQ_SA_31	pos	pos	2.08E+04	pos	pos	pos	pos
UQ_SA_32	neg	neg	4.26E+03	pos	pos	pos	neg
UQ_SA_33	neg	neg	3.03E+03	pos	pos	pos	neg
UQ_SA_34	neg	neg	2.14E+02	pos	neg	pos	neg
UQ_SA_35	neg	pos	2.79E+03	pos	pos	pos	neg
UQ_SA_36	neg	pos	1.12E+03	pos	pos	neg	neg
UQ_SA_37	pos	pos	4.01E+03	pos	pos	pos	pos
UQ_SA_38	neg	neg	4.42E+03	pos	pos	neg	neg
UQ_SA_39	neg	pos	4.42E+04	pos	pos	neg	neg
UQ_SA_40	pos	pos	1.47E+03	pos	pos	pos	pos
UQ_SA_41	pos	pos	6.95E+03	pos	pos	pos	neg
UQ_SA_42	neg	pos	3.45E+02	pos	pos	pos	neg

UQ_SA_43	pos	pos	1.75E+03	pos	pos	pos	pos
UQ_SA_44	pos	pos	8.71E+03	pos	pos	neg	pos
UQ_SA_45	pos	pos	1.26E+03	pos	pos	pos	pos
UQ_SA_46	neg	neg	2.35E+04	pos	pos	pos	neg
UQ_SA_47	neg	neg	3.65E+02	pos	pos	pos	neg
UQ_SA_48	neg	neg	4.95E+03	pos	pos	pos	neg
UQ_SA_49	neg	pos	1.36E+04	pos	pos	pos	pos
UQ_SA_50	neg	neg	9.41E+03	pos	pos	neg	neg
UQ_SA_51	pos	pos	1.92E+03	pos	pos	pos	pos
UQ_SA_54	pos	pos	2.22E+03	pos	pos	pos	pos
UQ_SA_55	pos	pos	6.26E+03	pos	pos	pos	pos
UQ_SA_56	pos	pos	7.99E+03	pos	pos	pos	pos
UQ_SA_58	pos	pos	6.35E+03	pos	pos	pos	pos
UQ_SA_59	neg	pos	9.53E+02	pos	pos	pos	neg
UQ_SA_61	pos	pos	2.16E+04	pos	pos	pos	pos
UQ_SA_63	neg	neg	9.11E+03	pos	pos	neg	neg
UQ_SA_64	pos	pos	6.71E+03	pos	pos	pos	pos
UQ_SA_65	pos	pos	9.81E+03	pos	pos	pos	pos
UQ_SA_66	neg	neg	5.05E+04	pos	neg	neg	pos
UQ_SA_67	pos	pos	1.07E+04	pos	pos	pos	pos
UQ_SA_68	pos	pos	8.03E+03	pos	pos	pos	pos
UQ_SA_69	neg	neg	2.46E+03	pos	pos	pos	neg
UQ_SA_70	neg	neg	4.48E+03	pos	pos	pos	neg
UQ_SA_71	neg	neg	6.36E+02	pos	pos	pos	neg
UQ_SA_72	neg	pos	7.63E+03	pos	pos	pos	neg
UQ_SA_73	neg	pos	5.38E+02	pos	pos	pos	neg
UQ_SA_74	neg	pos	1.25E+02	pos	pos	pos	neg
UQ_SA_75	pos	pos	6.04E+03	pos	pos	pos	pos
UQ_SA_76	pos	pos	1.09E+04	pos	pos	pos	pos
UQ_SA_77	neg	neg	1.60E+03	pos	pos	pos	neg
UQ_SA_78	pos	pos	1.28E+04	pos	pos	pos	pos
UQ_SA_79	neg	neg	1.35E+03	pos	pos	pos	neg
UQ_SA_80	neg	pos	2.13E+03	pos	pos	pos	neg
UQ_SA_81	neg	neg	neg	neg	neg	neg	neg
UQ_SA_82	neg	pos	1.65E+03	pos	pos	pos	neg
UQ_SA_83	pos	pos	8.71E+04	pos	pos	pos	neg
UQ_SA_84	neg	pos	6.16E+02	pos	pos	pos	neg
UQ_SA_85	neg	pos	2.75E+02	pos	pos	pos	neg
UQ_SA_86	pos	pos	4.59E+03	pos	pos	pos	pos
UQ_SA_87	neg	neg	5.29E+02	pos	pos	pos	neg
UQ_SA_88	neg	pos	8.31E+02	pos	pos	pos	neg
UQ_SA_89	pos	pos	2.97E+03	pos	pos	pos	pos
UQ_SA_90	neg	pos	8.06E+02	pos	pos	pos	neg
UQ_SA_91	neg	pos	1.06E+01	pos	pos	neg	neg

UQ_SA_92	pos	pos	9.45E+03	pos	pos	pos	pos
UQ_SA_93	neg	pos	2.15E+01	pos	pos	pos	neg
UQ_SA_94	neg	pos	1.44E+02	pos	pos	pos	neg
UQ_SA_95	neg	pos	2.32E+02	pos	pos	pos	neg
UQ_SA_96	neg	pos	9.96E+02	pos	pos	pos	neg
UQ_SA_97	neg	pos	2.30E+02	pos	pos	pos	neg
UQ_SA_98	neg	pos	9.11E+02	pos	pos	pos	neg
UQ_SA_99	neg	pos	2.47E+01	pos	pos	neg	neg
UQ_SA_100	pos	pos	1.65E+04	pos	pos	pos	pos
UQ_SA_101	neg	neg	4.63E+02	pos	neg	neg	neg
UQ_SA_102	neg	pos	8.20E+03	pos	pos	pos	pos
UQ_SA_103	neg	pos	1.11E+02	pos	pos	pos	neg
UQ_SA_104	neg	pos	2.81E+02	pos	pos	pos	neg
UQ_SA_105	neg	pos	1.97E+02	pos	pos	pos	neg

pos = positive; neg = negative; N/A = not available to test. P number is based on primer location depicted in figure 1

				RNA				
Env			any env	Gag	pol			any pol (real time+conventional)
P7	P6	P9			P3	P4	copies of viral load/ ml based on pol qRT-PCR	
pos	pos	pos	pos	N/A	N/A	N/A	N/A	N/A
pos	pos	pos	pos	neg	neg	neg	1.29E+05	pos
pos	pos	pos	pos	neg	neg	neg	3.46E+05	pos
pos	pos	neg	pos	N/A	N/A	N/A	N/A	N/A
pos	pos	pos	pos	pos	pos	NR	9.66E+09	pos
neg	neg	neg	neg	neg	neg	neg	neg	neg
pos	pos	pos	pos	N/A	N/A	N/A	N/A	N/A
pos	pos	pos	pos	neg	neg	neg	neg	neg
pos	pos	pos	pos	neg	neg	neg	neg	neg
neg	pos	neg	pos	neg	neg	neg	neg	neg
neg	neg	neg	neg	neg	neg	neg	neg	neg
pos	pos	neg	pos	neg	neg	neg	neg	neg
pos	pos	pos	pos	pos	pos	NR	1.64E+09	pos
pos	pos	pos	pos	pos	pos	NR	8.62E+08	pos
pos	pos	pos	pos	pos	pos	NR	4.34E+10	pos
pos	pos	neg	pos	neg	neg	neg	6.43E+04	pos
pos	neg	neg	pos	N/A	N/A	N/A	N/A	N/A
pos	neg	pos	pos	N/A	N/A	N/A	N/A	N/A
neg	pos	pos	pos	neg	neg	neg	neg	neg
pos	pos	pos	pos	neg	neg	neg	3.50E+05	pos
neg	pos	pos	pos	N/A	N/A	N/A	N/A	N/A
pos	pos	pos	pos	neg	neg	neg	neg	neg
pos	pos	pos	pos	N/A	N/A	N/A	N/A	N/A
pos	pos	pos	pos	N/A	N/A	N/A	N/A	N/A
pos	pos	pos	pos	N/A	N/A	N/A	N/A	N/A
pos	pos	pos	pos	N/A	N/A	N/A	N/A	N/A
pos	pos	pos	pos	N/A	N/A	N/A	N/A	N/A
pos	pos	pos	pos	N/A	N/A	N/A	N/A	N/A
pos	pos	pos	pos	N/A	N/A	N/A	N/A	N/A
pos	pos	pos	pos	N/A	N/A	N/A	N/A	N/A
pos	neg	neg	pos	N/A	N/A	N/A	N/A	N/A
pos	pos	neg	pos	N/A	N/A	N/A	N/A	N/A
pos	pos	neg	pos	N/A	N/A	N/A	N/A	N/A
pos	pos	pos	pos	N/A	N/A	N/A	N/A	N/A
pos	pos	neg	pos	neg	neg	neg	3.96E+04	pos
pos	pos	neg	pos	N/A	N/A	N/A	N/A	N/A
pos	pos	pos	pos	neg	neg	neg	8.48E+04	pos
pos	pos	pos	pos	neg	neg	neg	neg	neg
pos	pos	neg	pos	N/A	N/A	N/A	N/A	N/A

pos	pos	pos	pos	neg	neg	pos	1.59E+06	pos
pos	pos	pos	pos	N/A	N/A	N/A	N/A	N/A
pos	pos	pos	pos	N/A	N/A	N/A	N/A	N/A
pos	pos	neg	pos	N/A	N/A	N/A	N/A	N/A
pos	pos	neg	pos	N/A	N/A	N/A	N/A	N/A
pos	pos	pos	pos	N/A	N/A	N/A	N/A	N/A
pos	pos	pos	pos	N/A	N/A	N/A	N/A	N/A
pos	pos	neg	pos	N/A	N/A	N/A	N/A	N/A
pos	pos	pos	pos	neg	neg	neg	1.89E+05	pos
pos	pos	pos	pos	neg	neg	neg	neg	neg
pos	pos	pos	pos	pos	pos	NR	1.38E+09	pos
pos	pos	pos	pos	neg	neg	neg	2.55E+05	pos
pos	pos	pos	pos	neg	neg	neg	6.36E+04	pos
pos	pos	neg	pos	neg	neg	neg	2.28E+04	pos
pos	pos	pos	pos	neg	neg	neg	6.19E+05	pos
pos	pos	neg	pos	neg	neg	neg	neg	neg
pos	pos	pos	pos	N/A	N/A	N/A	N/A	N/A
pos	pos	pos	pos	N/A	N/A	N/A	N/A	N/A
pos	neg	pos	pos	neg	neg	neg	8.54E+04	pos
pos	pos	pos	pos	neg	neg	neg	1.85E+05	pos
pos	pos	pos	pos	N/A	N/A	N/A	N/A	N/A
pos	pos	pos	pos	N/A	N/A	N/A	N/A	N/A
pos	pos	neg	pos	neg	neg	neg	neg	neg
pos	pos	neg	pos	neg	neg	neg	neg	neg
pos	pos	neg	pos	N/A	N/A	N/A	N/A	N/A
pos	pos	pos	pos	N/A	N/A	N/A	N/A	N/A
pos	pos	pos	pos	N/A	N/A	N/A	N/A	N/A
pos	pos	pos	pos	N/A	N/A	N/A	N/A	N/A
pos	pos	pos	pos	N/A	N/A	N/A	N/A	N/A
pos	pos	neg	pos	N/A	N/A	N/A	N/A	N/A
pos	pos	pos	pos	N/A	N/A	N/A	N/A	N/A
pos	pos	neg	pos	N/A	N/A	N/A	N/A	N/A
pos	pos	neg	pos	N/A	N/A	N/A	N/A	N/A
neg	neg	neg	neg	N/A	N/A	N/A	N/A	N/A
pos	pos	neg	pos	neg	neg	neg	neg	neg
pos	pos	neg	pos	neg	neg	neg	neg	neg
pos	pos	neg	pos	neg	neg	neg	neg	neg
pos	pos	pos	pos	neg	neg	neg	neg	neg
pos	pos	pos	pos	N/A	N/A	N/A	N/A	N/A
pos	pos	neg	pos	neg	neg	neg	4.38E+04	pos
pos	pos	pos	pos	N/A	N/A	N/A	N/A	N/A
pos	pos	pos	pos	N/A	N/A	N/A	N/A	N/A
pos	pos	pos	pos	neg	neg	neg	neg	neg
pos	pos	neg	pos	N/A	N/A	N/A	N/A	N/A

pos	pos	pos	pos	neg	neg	neg	4.80E+04	pos
pos	pos	pos	pos	N/A	N/A	N/A	N/A	N/A
pos	pos	pos	pos	N/A	N/A	N/A	N/A	N/A
pos	pos	pos	pos	N/A	N/A	N/A	N/A	N/A
pos	pos	pos	pos	N/A	N/A	N/A	N/A	N/A
pos	pos	neg	pos	N/A	N/A	N/A	N/A	N/A
pos	pos	neg	pos	N/A	N/A	N/A	N/A	N/A
pos	pos	pos	pos	N/A	N/A	N/A	N/A	N/A
pos	pos	pos	pos	neg	neg	neg	neg	neg
pos	neg	neg	pos	N/A	N/A	N/A	N/A	N/A
pos	pos	pos	pos	N/A	N/A	N/A	N/A	N/A
pos	neg	neg	pos	neg	neg	neg	neg	neg
pos	pos	pos	pos	N/A	N/A	N/A	N/A	N/A
pos	pos	pos	pos	N/A	N/A	N/A	N/A	N/A

Env				any env
P8	P7	P6	P9	
N/A	N/A	N/A	N/A	N/A
neg	pos	pos	neg	pos
neg	pos	pos	neg	pos
N/A	N/A	N/A	N/A	N/A
pos	pos	pos	pos	pos
neg	neg	neg	neg	neg
N/A	N/A	N/A	N/A	N/A
neg	neg	neg	neg	neg
neg	pos	pos	neg	pos
neg	neg	neg	neg	neg
neg	neg	neg	neg	neg
neg	neg	neg	neg	neg
pos	pos	pos	pos	pos
pos	pos	pos	pos	pos
pos	pos	pos	pos	pos
neg	neg	neg	neg	neg
N/A	N/A	N/A	N/A	N/A
N/A	N/A	N/A	N/A	N/A
neg	neg	neg	neg	neg
neg	neg	neg	neg	neg
N/A	N/A	N/A	N/A	N/A
neg	neg	neg	neg	neg
N/A	N/A	N/A	N/A	N/A
N/A	N/A	N/A	N/A	N/A
N/A	N/A	N/A	N/A	N/A
N/A	N/A	N/A	N/A	N/A
N/A	N/A	N/A	N/A	N/A
N/A	N/A	N/A	N/A	N/A
N/A	N/A	N/A	N/A	N/A
N/A	N/A	N/A	N/A	N/A
N/A	N/A	N/A	N/A	N/A
N/A	N/A	N/A	N/A	N/A
N/A	N/A	N/A	N/A	N/A
N/A	N/A	N/A	N/A	N/A
neg	neg	neg	neg	neg
N/A	N/A	N/A	N/A	N/A
neg	neg	neg	neg	neg
neg	neg	neg	neg	neg
N/A	N/A	N/A	N/A	N/A

neg	pos	pos	neg	pos
N/A	N/A	N/A	N/A	N/A
N/A	N/A	N/A	N/A	N/A
N/A	N/A	N/A	N/A	N/A
N/A	N/A	N/A	N/A	N/A
N/A	N/A	N/A	N/A	N/A
N/A	N/A	N/A	N/A	N/A
N/A	N/A	N/A	N/A	N/A
neg	neg	neg	neg	neg
neg	neg	neg	neg	neg
pos	pos	pos	pos	pos
neg	neg	neg	neg	neg
neg	neg	pos	neg	pos
neg	neg	neg	neg	neg
neg	neg	neg	neg	neg
neg	neg	neg	neg	neg
N/A	N/A	N/A	N/A	N/A
N/A	N/A	N/A	N/A	N/A
neg	neg	neg	neg	neg
neg	neg	neg	neg	neg
N/A	N/A	N/A	N/A	N/A
N/A	N/A	N/A	N/A	N/A
neg	neg	neg	neg	neg
neg	neg	neg	neg	neg
N/A	N/A	N/A	N/A	N/A
N/A	N/A	N/A	N/A	N/A
N/A	N/A	N/A	N/A	N/A
N/A	N/A	N/A	N/A	N/A
N/A	N/A	N/A	N/A	N/A
N/A	N/A	N/A	N/A	N/A
N/A	N/A	N/A	N/A	N/A
N/A	N/A	N/A	N/A	N/A
N/A	N/A	N/A	N/A	N/A
N/A	N/A	N/A	N/A	N/A
neg	neg	neg	neg	neg
neg	neg	neg	neg	neg
neg	neg	neg	neg	neg
neg	neg	neg	neg	neg
N/A	N/A	N/A	N/A	N/A
neg	neg	neg	neg	neg
N/A	N/A	N/A	N/A	N/A
N/A	N/A	N/A	N/A	N/A
neg	neg	neg	neg	neg
N/A	N/A	N/A	N/A	N/A

neg	neg	neg	neg	neg
N/A	N/A	N/A	N/A	N/A
N/A	N/A	N/A	N/A	N/A
N/A	N/A	N/A	N/A	N/A
N/A	N/A	N/A	N/A	N/A
N/A	N/A	N/A	N/A	N/A
N/A	N/A	N/A	N/A	N/A
N/A	N/A	N/A	N/A	N/A
neg	neg	neg	neg	neg
N/A	N/A	N/A	N/A	N/A
N/A	N/A	N/A	N/A	N/A
neg	neg	neg	neg	neg
N/A	N/A	N/A	N/A	N/A
N/A	N/A	N/A	N/A	N/A
